# The Effect of κ-Carrageenan on Porcine Sperm Cryo-Survival

**DOI:** 10.3390/ani14091387

**Published:** 2024-05-06

**Authors:** Areeg Almubarak, Eunji Kim, Il-Jeoung Yu, Hanseul Park, Yubyeol Jeon

**Affiliations:** 1 Department of Theriogenology and Reproductive Biotechnology, College of Veterinary Medicine, Jeonbuk National University, Iksan 54596, Republic of Korea or areegalmubarak@gmail.com (A.A.); dmswl2570@naver.com (E.K.); iyu@jbnu.ac.kr (I.-J.Y.); 2 Department of Veterinary Medicine and Surgery, College of Veterinary Medicine, Sudan University of Science and Technology, Hilat Kuku, P.O. Box 204, Khartoum North 11111, Sudan; 3 Laboratory of Molecular Genetics, College of Pharmacy, Chungbuk National University, Cheongju 28160, Republic of Korea

**Keywords:** κ-Carrageenan, boar, cryopreservation, cryoinjury, artificial insemination, polysaccharides

## Abstract

**Simple Summary:**

Boar sperm is highly sensitive to reactive oxygen species (ROS) during the freezing–thawing process. κ-Carrageenan is a sulfated polysaccharide from seaweed reported to possess substantial antioxidant activities. However, the influence of κ-Carrageenan on porcine sperm cryo-survival remains to be clarified. Therefore, different concentrations of 0 (control), 0.2, 0.4, 0.6, and 0.8 mg/mL κ-Carrageenan were added to the freezing diluent. Sperm kinematics were assessed (CASA software) immediately after thawing (AT) and post-incubation for 120 min. Viability, acrosome integrity, lipid peroxidation, and intracellular caspase activities were measured AT using SYBR-14/PI, PSA/FITC, the MDA assay, and a commercial kit, respectively. The results indicated that the addition of κ-Carrageenan to the extender could enhance the quality of frozen–thawed (FT) boar sperm through its influence on membrane stability, mitochondrial potential, and reducing oxidative stress. Furthermore, 0.2 mg/mL κ-Carrageenan was revealed to be the optimal concentration for FT sperm quality.

**Abstract:**

κ-Carrageenan is a sulfated polysaccharide from red seaweed with substantial antioxidant activities. This study aimed to investigate the effect of κ-Carrageenan treatment on frozen–thawed (FT) porcine semen quality. Therefore, the spermatozoa were diluted and cryopreserved in a freezing extender supplemented with 0 (control), 0.2, 0.4, 0.6, and 0.8 mg/mL κ-Carrageenan. Sperm kinematics were assessed immediately after thawing (AT) and post-incubation for 120 min. The viability, acrosome integrity, lipid peroxidation, mitochondrial membrane potential (MMP), and intracellular caspase activity were measured AT. The results indicated that 0.2 mg/mL κ-Carrageenan increased total and progressive motility AT and post-incubation for 120 min (*p* < 0.05). Moreover, the viable sperm percentage and MMP after 0.2 mg/mL treatment were higher than those after control and other κ-Carrageenan concentration treatments. The proportion of acrosome-intact spermatozoa was significantly higher after 0.2 and 0.4 mg/mL κ-Carrageenan treatment than that after control and other κ-Carrageenan concentration treatments. The intracellular caspase activity was not significantly different among the experimental groups. However, the MDA concentration after 0.2 mg/mL κ-Carrageenan treatment was lower (*p* < 0.05) than that after the control treatment. Taken together, adding κ-Carrageenan to the porcine semen freezing extender improved the FT sperm quality mainly by influencing membrane stability and protecting against oxidative stress.

## 1. Introduction

Sperm cryopreservation is considered the most efficient approach for the long-term storage of spermatozoa and genetic material preservation in most domestic animals [1,2]. However, the use of frozen–thawed (FT) semen for commercial swine artificial insemination (AI) is limited because of its lower fertility outcomes than that of extended fresh semen. As boar sperm are highly susceptible to cold shock, the viability of the sperm cells is substantially reduced when exposed to temperatures below 15 °C [3,4]. Therefore, continuous efforts are being made to optimize the FT protocols. The roles of additives, such as sugars [5,6], proteins [7], cryoprotectants [8,9], and antioxidants [10,11], have also been studied. Despite all such efforts, this procedure has not yet been as effective in swine as it has in other species, and only 1% of AI is accompanied by cryopreserved sperm [12,13].

Endogenous antioxidants are insufficient to protect the spermatozoa from oxidative stress during cryopreservation. The FT protocol reduces seminal plasma enzymatic and non-enzymatic antioxidant content owing to reconstitution processes before storage [14,15]. Furthermore, the physical and chemical conditions to which spermatozoa are exposed during cryopreservation trigger reactive oxygen species (ROS) accumulation. Excess ROS generation during FT is detrimental to sperm motility and fertilization ability [16]. Moreover, the high amount of unsaturated fatty acids in porcine spermatozoa makes them highly sensitive to lipid peroxidation and fatty acid disruption in the plasma membrane [17,18]. Therefore, antioxidant supplementation is a reasonable strategy for mitigating the negative effects of cryopreservation and ultimately improving sperm quality and fertility. Indeed, the inclusion of antioxidants such as a-tocopherol [19], glutathione [20], L-cysteine [21], and rosmarinic acid [22] during sperm cryopreservation has been shown to benefit boar sperm after freezing–thawing.

Carrageenan is a natural sulfated polysaccharide extracted from the Rhodophyceae family of red seaweeds [23,24]. It has been used as a food additive and has several potential pharmaceutical applications, such as antioxidant, anticancer, and immunomodulatory activities [23,25,26]. Furthermore, κ-Carrageenan improves dog [27] and rooster [28] sperm cryo-survival through diminishing intracellular ROS, modulating apoptosis, and upregulating endogenous antioxidant enzymatic activities. Despite these beneficial effects of κ-Carrageenan, it has not been used for porcine semen cryopreservation. This study aimed to explore the effect of different κ-Carrageenan concentrations in the freezing extender on post-thaw semen quality parameters of porcine spermatozoa.

## 2. Materials and Methods

### 2.1. Reagents and Extender Preparation

Equex Paste STM was purchased from Minitube (Munich, Germany). The solutions were prepared using high-purity water procured from ProGen (Genetrone Biotech, Jeonju, Korea). All other chemicals used in this study were obtained from Sigma-Aldrich (St. Louis, MO, USA) unless otherwise indicated.

The freezing extender used in this experiment was BF5 extender [29], which is composed of 12 g/L TES, 2 g/L Trizma Base, 32 g/L D(+) glucose, 0.7% (*v*/*v*) OEP (Equex), 0.02 g/L gentamycin sulfate, and 20% (*v*/*v*) egg yolk. Extender (2) was composed of extender 1 and 4% (*v*/*v*) glycerol. Beltsville thawing solution (BTS) consists of 37 g/L D(+) glucose, 1.25 g/L Na-EDTA, 6 g/L sodium citrate dihydrate, 1.25 g/L sodium bicarbonate, 0.75 g/L potassium chloride, 0.6 g/L penicillin, and 1 g/L streptomycin [30].

### 2.2. Semen Collection

Semen was collected from Duroc boars (N = 8) of proven fertility belonging to a local livestock AI center (KPG, Gimje, Korea) using the gloved-hand method. Sperm-rich fractions of ejaculates with more than 75% motility and 80% morphologically normal spermatozoa were used in this study (N = 24) [20,30]. Eligible samples were diluted (2.5 × 10^9^ ± 0.5 spermatozoa/90 mL) in BTS. The diluted semen was cooled and maintained at 17 °C for shipment to the laboratory within 1 h.

### 2.3. Sperm Cryopreservation Process

Semen samples were cryopreserved using a modified two-step freezing protocol as described previously [31,32]. Briefly, the diluted semen was transferred to 15 mL centrifuge tubes and cooled at 17 °C for at least 2 h and later centrifuged at 2000 rpm for 7 min at 17 °C, whereupon the supernatant was discarded and the sperm pellet was resuspended in BTS. The sample was then centrifuged at 2000 rpm for 7 min at 17 °C. The supernatant was removed, and the sperm pellet was resuspended with extender 1 to a concentration of 2 × 10^8^ sperm/mL. The extended semen was cooled at 4 °C for 60 min and mixed with extender 2 (1:1 *v*/*v*). Samples were then loaded into 0.5 mL straws (imv Technologies, Laigle, France), sealed, and incubated at 4 °C for 25 min. The straws were placed horizontally in a polystyrene box 4 cm above liquid nitrogen vapor for 20 min and then plunged in liquid nitrogen for storage.

### 2.4. Evaluation of FT Sperm Kinematics

FT sperm kinematics was assessed immediately after thawing (AT) and post-incubation for 120 min using Sperm Class Analyzer (SCA) software (Version 5.1; Microptic, Barcelona, Spain). Briefly, the samples were thawed at 38 °C for 25 s and then diluted in BTS (1:4 *v*/*v*). Then, 2 μL of diluted semen were loaded into a pre-warmed counting chamber (Leja, Nieuw Vennep, The Netherlands). Samples were kept at 24 °C for 2 h before being analyzed again. For each analysis, at least 1500 spermatozoa were tested using standard settings (38 °C, 60 frames/s). The percentages of total motile spermatozoa (TM, %), progressive motile spermatozoa (PM, %), and mucus penetration (MP, %) were determined. The kinematic parameters measured for each sperm included curvilinear velocity (VCL, μm/s), straight-line velocity (VSL, μm/s), average path velocity (VAP, μm/s), and amplitude of lateral head displacement (ALH, μm). This experiment was repeated six times.

### 2.5. Evaluation of Sperm Viability

The viability of FT sperm was evaluated using the LIVE/DEAD^®^ Sperm Viability Kit (ThermoFisher, Waltham, MA, USA) according to the previously described method [33]. In short, 5 μL SYBR-14 was added to 50 μL sperm suspension and incubated for 5 min in the dark. Then, 5 μL PI was added, and the mixture was incubated again for 5 min. Smears from each group were observed under a fluorescence microscope (Axio, Carl Zeiss, Oberkochen, Baden-Württemberg, Germany) and classified as live (green fluorescent) or dead (red fluorescent) spermatozoa. This experiment was repeated six times.

### 2.6. Assessment of Acrosome Status

Sperm acrosome integrity was evaluated using a previously described fluorescence staining method [33]. Briefly, thin smears were prepared from the FT semen of each group and air-dried. The samples were fixed with methanol and stained with fluorescein isothiocyanate (FITC)-labeled *Pisum sativum* agglutinin (PSA). The stained smears were covered with Parafilm, incubated for 20 min, rinsed with distilled water, and dried. At least 200 sperm per sample were assessed under a fluorescence microscope (Axio, Carl Zeiss) to determine the percentage of sperm with intact and reacted acrosomes. This experiment was repeated six times.

### 2.7. Evaluation of Sperm Mitochondrial Activity

Rhodamine 123 (Rh123) and PI staining were used to measure FT sperm mitochondrial activity, as described previously [34]. Briefly, 5 μL R123 solution (0.01 mg/mL) and 5 μL PI were mixed with 250 μL diluted sample and incubated in the dark at 37 °C for 15 min. Then, sperm smears were prepared and examined under a fluorescence microscope (Axio, Carl Zeiss). PI-negative and Rh123-positive sperm were identified as live sperm with high MMP.

### 2.8. Measurement of Intracellular Caspase Activities

The Casp-GLOW Red Active Caspase Staining Kit (Bio Vision, Milpitas, CA, USA) was used to assess the intracellular caspase (caspase-1, -3, -4, -5, -7, -8, and -9) activity in FT sperm as described previously [35] with some modifications. Briefly, FT semen was diluted (1:4) in phosphate-buffered saline. Then, 300 μL sperm suspension containing 1 μL Red–VAD–FMK solution was incubated at 38 °C for 40 min. Sperm were washed by centrifugation at 1500 rpm for 3 min in 500 μL assay–wash buffer. The sperm sediment was resuspended to a final concentration of 2 × 10^7^ sperm/mL in an assay–wash buffer before being assessed under a fluorescence microscope. Sperm with bright red fluorescence in the midpiece and tail were considered positive for caspase activity. Approximately 150 sperm were analyzed per trial in each experimental group.

### 2.9. Measurement of Lipid Peroxidation

The degree of lipid peroxidation in FT samples was evaluated using the Lipid Peroxidation (MDA) Colorimetric/Fluorometric Assay Kit (BioVision, Mountain View, CA, USA) following the kit’s instructions. Briefly, FT semen was centrifuged at 1500 rpm for 3 min, and the supernatant was discarded. The number of sperm cells was adjusted to 2 × 10^7^/mL. The sperm pellets were dissolved in 300 μL MDA Lysis Buffer, centrifuged (13,000× *g*, 10 min), and 200 μL supernatant was incubated with 600 μL TBA reagent for 60 min at 95 °C. Next, the tubes were cooled to room temperature for 10 min in an ice bath, and the absorbance was measured at 532 nm using a spectrophotometric microplate reader. This experiment was repeated six times, and the resultant MDA concentration is given as nmol/2 × 10^7^ sperm.

### 2.10. Statistical Analysis

Each experiment was repeated four times, unless otherwise mentioned in the above sections, and the results were averaged. Data were analyzed using the SAS software, version 8.4 (SAS Institute Inc., Cary, NC, USA). The averaged data were compared using a one-way analysis of variance, followed by Duncan’s multiple range test. The results are shown as mean ± standard error, and *p* < 0.05 was considered significant.

## 3. Results

### 3.1. Effects of κ-Carrageenan on Post-Thawing Kinematic Parameters

**Figure 1:**
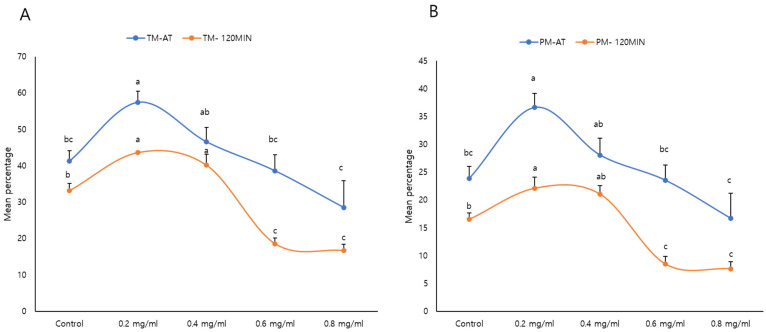
Effect of κ-Carrageenan supplementation in extenders on post-thawing sperm kinematic patterns: TM: total motility (**A**) and PM: progressive motility (**B**) at thawing (AT) and post-incubation for 120 min. The letters above the bar represent significant differences between groups (*p* < 0.05). Error bars show the standard error of the mean.

The percentage of total and progressive motile sperm was significantly higher in the 0.2 mg/mL κ-Carrageenan-treated group than those in the control and 0.6 and 0.8 mg/mL κ-Carrageenan-treated groups (*p* < 0.05) at thawing (AT) and post-incubation for 120 min. Further, 0.2 and 0.4 mg/mL κ-Carrageenan treatment increased the percentage of motile spermatozoa after thawing for 120 min. However, the 0.8 mg/mL κ-Carrageenan-treated group exhibited significantly lower kinematic patterns than the control and other κ-Carrageenan-treated groups (Figure 1, Table 1).

### 3.2. Effects of κ-Carrageenan on FT Sperm Viability

**Figure 2:**
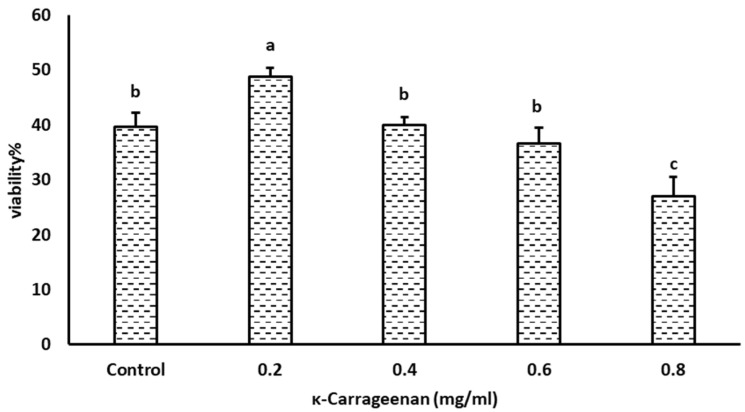
Effect of κ-Carrageenan supplementation in extenders on the post-thaw viability of boar spermatozoa. Different alphabets above the bar mean significant differences (*p* < 0.05) among treatments.

The proportion of viable spermatozoa was significantly higher in 0.2 mg/mL κ-Carrageenan-treated sperm than that in control and other κ-Carrageenan-treated sperm (*p* < 0.05, Figure 2). However, there were no significant differences between the control, 0.4, and 0.6 mg/mL κ-Carrageenan-treated groups. Further, the 0.8 mg/mL κ-Carrageenan-treated group had a lower proportion of viable spermatozoa than the other groups (*p* < 0.05).

### 3.3. Effects of κ-Carrageenan on Post-Thawing Acrosome Integrity

**Figure 3:**
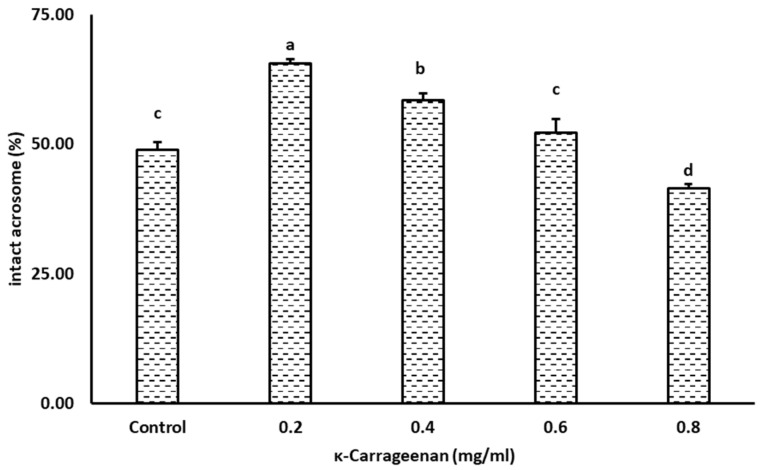
Effect of κ-Carrageenan treatment in extenders on post-thaw acrosome integrity of boar spermatozoa. Different alphabets above the bar indicate statistical differences (*p* < 0.05) among treatments.

The percentage of acrosome-intact spermatozoa was significantly higher in the 0.2 and 0.4 mg/mL κ-Carrageenan-treated groups than that in other groups (Figure 3). Moreover, the 0.2 mg/mL κ-Carrageenan-treated group had a higher percentage of acrosome-intact spermatozoa than the 0.4 mg/mL κ-Carrageenan-treated group (*p* < 0.05). Interestingly, there was no difference in the percentage of acrosome-intact spermatozoa between the 0.6 mg/mL κ-Carrageenan-treated group and the control. However, it was significantly lower in the 0.8 mg/mL κ-Carrageenan-treated group than in the other groups.

### 3.4. Effects of κ-Carrageenan on FT Sperm Mitochondrial Activity

**Figure 4:**
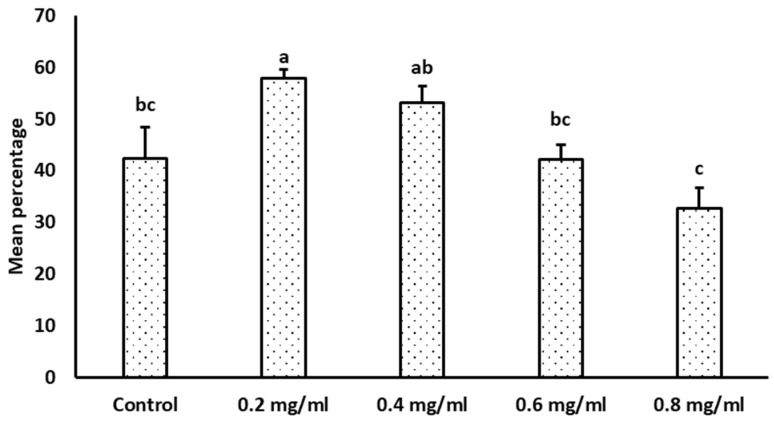
Effect of κ-Carrageenan treatment in extenders on the post-thaw mitochondrial membrane potential of boar spermatozoa. Results are given as the mean percentage ± standard error (SE). Error bars indicate the standard error of the mean. Different alphabets above the bar represent statistical differences (*p* < 0.05) among treatments.

The proportion of FT sperm with active mitochondria was significantly higher in 0.2 and 0.4 mg/mL κ-Carrageenan-treated sperm than in the control (*p* < 0.05). However, it was not significantly different between the control and 0.6 mg/mL κ-Carrageenan-treated groups. Furthermore, the 0.8 mg/mL κ-Carrageenan-treated group had a lower proportion (*p* < 0.05) of active mitochondria than the other groups (Figure 4).

### 3.5. Effects of κ-Carrageenan on Intracellular Caspase Activities

**Figure 5:**
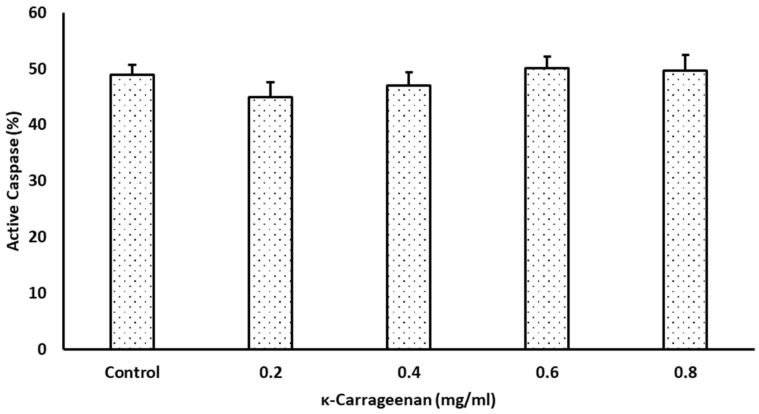
Effect of κ-Carrageenan supplementation in extender on intracellular caspase activities of frozen–thawed boar spermatozoa. Results are given as the mean percentage ± standard error (SE). Error bars indicate the standard error of the mean (*p* > 0.05).

In contrast to the improved FT sperm quality parameters after κ-Carrageenan treatment, the intracellular caspase activities in the presence or absence of κ-Carrageenan did not differ statistically (*p* > 0.05; Figure 5).

### 3.6. Effects of κ-Carrageenan on Lipid Peroxidation

**Figure 6:**
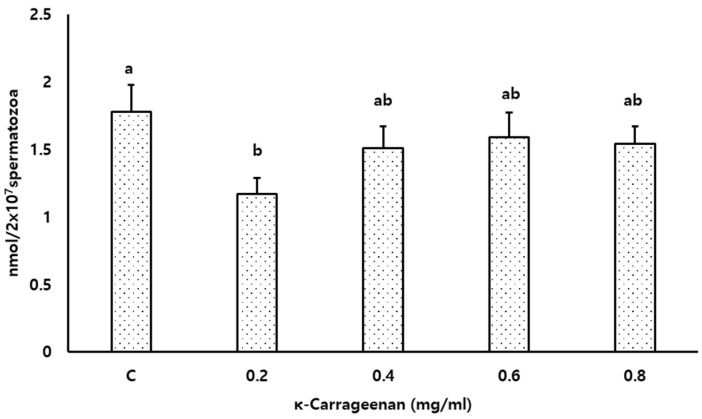
Effect of κ-Carrageenan supplementation in extenders on lipid peroxidation (MDA levels) of frozen–thawed boar spermatozoa. Different alphabets above the bar indicate significant differences (*p* < 0.05) among treatments.

As shown in Figure 6, MDA concentrations were lower (*p* < 0.05) in the 0.2 mg/mL κ-Carrageenan-treated group than those in the control group.

## 4. Discussion

Freezing–thawing results in a deterioration of sperm motility and fertility due to ROS accumulation and oxidative damage. The consequences of cryoinjury include impaired passage and poor survival of spermatozoa in the female reproductive tract [16,36]. Thus, strategies for ameliorating stress by including antioxidants in extenders for sperm cryopreservation have been investigated. In this study, we evaluated the effects of κ-Carrageenan supplementation in freezing extenders on post-thaw sperm quality. The results indicated that adding κ-Carrageenan to the semen extender improves post-thaw semen quality by enhancing motion parameters, maintaining membranous integrity and mitochondrial functionality, and reducing oxidative stress in boar spermatozoa.

κ-Carrageenan, a sulfated polysaccharide from seaweed, possesses antioxidant activities [23,37]. The mechanism through which κ-Carrageenan protects against oxidative stress is related to its chemical structure. The sulfate content of this algal polysaccharide is responsible for neutralizing free radicals, a process that has been thoroughly reviewed previously [37,38]. However, to the best of our knowledge, no previous study has reported the effects of κ-Carrageenan on cryopreserved boar sperm. κ-Carrageenan at concentrations of 0.2 mg/mL and 0.4 mg/mL seems to provide the best effect on sperm functionality after freezing–thawing. In the present study, spermatozoa treated with 0.2 mg/mL κ-Carrageenan showed improved kinematic parameters and maintained membranous integrity as well as mitochondrial potential. The importance of different sperm kinematic parameters and their correlation with fertilization capacity has been emphasized in previous studies [39,40]. Nevertheless, functional membranes and mitochondria are crucial for sperm motility, acrosome reactions, capacitation, and fertilization [41,42]. Freeze–thaw stress compromises the functional integrity of the plasma membrane due to ROS-mediated peroxidative damage. In addition, cryopreservation causes mitochondrial damage and consequently causes loss of sperm motility due to ATP depletion. MMP reflects mitochondrial activity and energy status and is correlated with sperm motility and viability in several species [43,44]. In this study, adding κ-Carrageenan to the extender improved the membrane integrity of FT boar spermatozoa. These findings are consistent with those of previous studies using canine [27] and rooster [28] semen samples. Such plant polysaccharide-promoted cryo-survival has been reported in boar [45] and human [46] spermatozoa.

Polyunsaturated fatty acids (PUFAs) play a fundamental role in membrane structure and function. However, a high content of PUFA makes the sperm membrane susceptible to lipid peroxidation in the presence of ROS [47,48]. Sperm cryopreservation is also associated with ROS accumulation, as ROS attacks and destroys vital macromolecules such as proteins, lipid membranes, and DNA, thus impairing sperm function [49,50]. Furthermore, dead and damaged sperm can trigger lipid peroxidation [36]. In this study, lipid peroxidation in sperm was significantly reduced in the 0.2 mg/mL κ-Carrageenan treated group than in the control. This finding concurs with the report of [28], which demonstrated the MDA-lowering effect following the treatment of rooster sperm freezing extender with κ-Carrageenan. Furthermore, the in vitro and in vivo antioxidant capacities of sulfated polysaccharides derived from seaweeds in different cell lines have been emphasized [38,51,52,53]. Taken together, adding κ-Carrageenan suppressed lipid peroxidation, protecting the plasma membrane, acrosome, and MMP during the FT process.

The presence of caspases in spermatozoa is one of the best cellular apoptosis markers, and their activation substantially influences the apoptotic pathway [54,55]. Sperm cryopreservation induces changes related to apoptosis, such as phosphatidylserine externalization as well as DNA fragmentation generation and exacerbation [56]. Indeed, DNA fragmentation is correlated with apoptotic cell death, decreased semen quality, and impaired fertilization potential [50,57]. In this study, κ-Carrageenan treatment during sperm cryopreservation did not inhibit caspase activity. These findings are in line with those of Zribi et al. (2012) and Thomson et al. (2009) [58,59], who suggested that the increase in sperm DNA damage during freeze–thaw cycles is mainly mediated through oxidative stress rather than the activation of caspases and apoptosis. However, oxidative stress can induce mitochondrial DNA damage and, in a loop manner, generate secondary ROS that activate stress response genes, eventually leading to apoptosis [57]. Furthermore, other mechanisms responsible for cryopreservation damage, including chemical and physical alterations to which spermatozoa are exposed during FT, could also disrupt the sperm structure [60,61]. Taken together, the results of this study point to the possibility of a caspase-independent apoptosis pathway; however, further studies on the appropriate apoptosis-inducing factors at different steps of the sperm freeze–thaw process would be helpful for clearly understanding and ultimately improving FT sperm quality.

## 5. Conclusions

In conclusion, supplementation of the extender with κ-Carrageenan improved the freezability of porcine spermatozoa. Adding 0.2 mg/mL κ-Carrageenan to the extender protects post-thaw sperm kinematics, membrane integrity, and lipid peroxidation. Therefore, 0.2 mg/mL κ-Carrageenan may be recommended for supplementation in a semen extender for boar semen cryopreservation.

## Figures and Tables

**Table 1:** Effects of κ-Carrageenan on post-thawing sperm kinematics.

Group (mg/mL)	MP (%)	VCL (µm/s)	VAP (µm/s)	VSL (µm/s)	ALH (µm)
Control–AT	12.77 ± 1.90 ^ab^	50.43 ± 2.98 ^ab^	38.67 ± 1.38 ^a^	31.13 ± 1.51 ^ab^	1.82 ± 0.15 ^ab^
Post 120 min	6.92 ± 1.91 ^a^	38.73 ± 4.73 ^a^	31.51 ± 4.68 ^a^	25.73 ± 5.15 ^a^	1.42 ± 0.15 ^a^
0.2–AT	18.69 ± 2.01 ^a^	56.03 ± 1.66 ^a^	43.67 ± 1.59 ^a^	34.41 ± 2.14 ^a^	1.96 ± 0.10 ^a^
Post 120 min	9.58 ± 3.55 ^a^	40.72 ± 5.88 ^a^	32.76 ± 5.63 ^a^	26.83 ± 5.94 ^a^	1.49 ± 0.16 ^a^
0.4–AT	15.37 ± 1.86 ^ab^	50.87 ± 1.51 ^ab^	40.26 ± 1.43 ^a^	32.46 ± 1.50 ^ab^	1.78 ± 0.07 ^ab^
Post 120 min	9.70 ± 1.60 ^a^	45.73 ± 6.75 ^a^	34.33 ± 3.63 ^a^	27.93 ± 4.39 ^a^	1.51 ± 0.06 ^a^
0.6–AT	11.56 ± 2.10 ^bc^	48.85 ± 1.56 ^b^	39.42 ± 1.91 ^a^	31.69 ± 2.40 ^ab^	1.65 ± 0.08 ^b^
Post 120 min	3.31 ± 0.94 ^a^	37.49 ± 4.93 ^a^	28.48 ± 3.89 ^a^	22.14 ± 3.96 ^a^	1.47 ± 0.18 ^a^
0.8–AT	6.42 ± 1.96 ^c^	43.03 ± 1.86 ^c^	33.39 ± 2.20 ^b^	26.25 ± 2.54 ^b^	1.58 ± 0.07 ^b^
Post 120 min	2.95 ± 1.11 ^a^	37.56 ± 4.06 ^a^	29.71 ± 3.65 ^a^	23.78 ± 4.34 ^a^	1.41 ± 0.09 ^a^

AT: at thawing. MP, mucus penetration; VCL, curvilinear velocity; VAP, average path velocity; VSL, straight-line velocity; ALH, amplitude of lateral head displacement. Values indicate the mean ± standard error of the mean (SEM). Different superscripts within the same column indicate significant differences (*p* < 0.05).

## Data Availability

The data supporting the findings of this study are available upon reasonable request from the corresponding author (Y.J.).
